# Untargeted metabonomic analysis of a cerebral stroke model in rats: a study based on UPLC–MS/MS

**DOI:** 10.3389/fnins.2023.1084813

**Published:** 2023-08-08

**Authors:** Dunbing Huang, Yihan Yang, Wei Song, Cai Jiang, Yuhao Zhang, Anren Zhang, Zhonghua Lin, Xiaohua Ke

**Affiliations:** ^1^Department of Rehabilitation Medicine, Shanghai Fourth People’s Hospital, School of Medicine, Tongji University, Shanghai, China; ^2^College of Rehabilitation Medicine, Fujian University of Traditional Chinese Medicine, Fuzhou, China; ^3^Shengli Clinical Medical College of Fujian Medical University, Fuzhou, China; ^4^Second Rehabilitation Department, Fujian Provincial Hospital, Fuzhou, China; ^5^Fujian Provincial Center for Geriatrics, Fujian Provincial Hospital, Fuzhou, China; ^6^Fujian Key Laboratory of Geriatrics Diseases, Fujian Provincial Hospital, Fuzhou, China; ^7^Department of Complementary Medicine, University of Johannesburg, Johannesburg, South Africa; ^8^Department of Rehabilitation Medicine, Nanjing Lishui District Hospital of Traditional Chinese medicine, Nanjing, China

**Keywords:** untargeted metabolomics, ischemic stroke, rats, tryptophan metabolism, metabolism

## Abstract

**Introduction:**

Brain tissue damage caused by ischemic stroke can trigger changes in the body’s metabolic response, and understanding the changes in the metabolic response of the gut after stroke can contribute to research on poststroke brain function recovery. Despite the increase in international research on poststroke metabolic mechanisms and the availability of powerful research tools in recent years, there is still an urgent need for poststroke metabolic studies. Metabolomic examination of feces from a cerebral ischemia–reperfusion rat model can provide new insights into poststroke metabolism and identify key metabolic pathways, which will help reveal diagnostic and therapeutic targets as well as inspire pathophysiological studies after stroke.

**Methods:**

We randomly divided 16 healthy adult pathogen-free male Sprague–Dawley (SD) rats into the normal group and the study group, which received middle cerebral artery occlusion/reperfusion (MCAO/R). Ultra-performance liquid chromatography–tandem mass spectrometry (UPLCMS/MS) was used to determine the identities and concentrations of metabolites across all groups, and filtered high-quality data were analyzed for differential screening and differential metabolite functional analysis.

**Results:**

After 1 and 14 days of modeling, compared to the normal group, rats in the study group showed significant neurological deficits (*p* < 0.001) and significantly increased infarct volume (day 1: *p* < 0.001; day 14: *p* = 0.001). Mass spectra identified 1,044 and 635 differential metabolites in rat feces in positive and negative ion modes, respectively, which differed significantly between the normal and study groups. The metabolites with increased levels identified in the study group were involved in tryptophan metabolism (*p* = 0.036678, *p* < 0.05), arachidonic acid metabolism (*p* = 0.15695), cysteine and methionine metabolism (*p* = 0.24705), and pyrimidine metabolism (*p* = 0.3413), whereas the metabolites with decreased levels were involved in arginine and proline metabolism (*p* = 0.15695) and starch and sucrose metabolism (*p* = 0.52256).

**Discussion:**

We determined that UPLC–MS/MS could be employed for untargeted metabolomics research. Moreover, tryptophan metabolic pathways may have been disordered in the study group. Alterations in the tryptophan metabolome may provide additional theoretical and data support for elucidating stroke pathogenesis and selecting pathways for intervention.

## 1. Introduction

Ischemic stroke is a clinical syndrome characterized by rapid onset of focal or global brain deficits lasting more than 24 h (Shen et al., 2023). Ischemic stroke condition manifests as motor, sensory, cognitive and speech dysfunction (Winstein et al., 2016) and places a heavy burden on the economy and society due to its high morbidity, mortality and disability rates (Bai et al., 2017). Stroke-induced brain damage is caused by a complex series of neuropathological events, mainly including oxidative stress, excitotoxicity, apoptosis and neuroinflammation (Kunz et al., 2010; Radenovic et al., 2020). The associated pathophysiological responses further leads to changes in the patient’s metabolism and alterations in metabolites.

The gut-brain axis (GBA) is a two-way communication network between the gastrointestinal tract and the central nervous system. After stroke, the GBA communicates from the brain to the gut (top-down signaling) and plays a regulatory role at several levels, directly or indirectly. Stroke impairs intestinal function, affecting intestinal motility (Singh et al., 2016; Jiang et al., 2021), subsequently altering intestinal flora (Chen et al., 2019; Lee et al., 2020), resulting in changes in flora metabolism and ultimately metabolites such as feces (Winek et al., 2016; Jiang et al., 2021; Huang et al., 2022). Therefore, the corresponding metabolic changes in the gut microbes are thought to be one of the hallmarks of stroke pathogenesis (Benakis et al., 2016; Zhu et al., 2021). In turn, gut microbiota dysbiosis appears to communicate with the brain through the microbial gut-brain axis (bottom-up signaling), thereby exacerbating the deleterious effects of stroke (Benakis et al., 2016; Singh et al., 2016). Studies have shown that short-chain fatty acids and indole derivatives produced by gut microbes, among others, can influence brain function by influencing energy metabolism, inflammation and neurotransmitter changes in the brain, making them functional preservers of the brain (Winek et al., 2016; Roth et al., 2021; Huang et al., 2022) (2, 27,714,645). Therefore, a growing amount of research supports the notion that gut microbes are significant regulators of GBA and drivers of amino acid metabolism (De Vadder et al., 2014; Fröhlich et al., 2016). Eventually, metabolites related to host–microbe interactions have become new targets for testing (Mu et al., 2016).

Metabolomics is one of the most widely used applications for tracking metabolites (Ma et al., 2012). This approach allows researchers to identify and screen for biomarkers. It also allows the study of typological and quantitative changes in endogenous metabolites, as well as their intrinsic relationship and broad patterns of change with physiological and pathological phenotypes following perturbations. Furthermore, by focusing on host–microbe interactions, metabolomics can identify metabolites (qualitatively and quantitatively) associated with these interactions and ultimately reveal the metabolic mechanisms underlying disease pathogenesis.Thus, metabolomics is uniquely positioned to help identify biomarkers specific to disease mechanisms (Bujak et al., 2016; Johnson et al., 2016). One of the research approaches in metabolomics is nontargeted metabolomics, which is used to analyze as many metabolites as possible without prior knowledge of these metabolites (Dunn et al., 2012). Metabolomics is also increasingly used in the course of clinical research in stroke because of its potential role in the discovery of biomarkers for the early diagnosis of ischemic stroke and the development of new therapeutic targets (Kimberly et al., 2013; Jové et al., 2014; Sidorov et al., 2019; Montaner et al., 2020).

The ultra-performance liquid chromatography (UPLC) employed in this study is the most widely used technique for metabolomics analysis with ultrahigh efficiency, ultrahigh separation, ultrahigh sensitivity and low consumption, and this method dominates the field of metabolomics (Barri and Dragsted, 2013; Yin et al., 2016; Ma et al., 2017). When coupled with mass spectrometry measures, such as quadrupole time of flight (Q-TOF), ESI (QQQ) or MS/MS (Q-Trap), UPLC offers significant advantages in qualitative as well as quantitative analysis. Su et al. (2016) investigated the protective effect of a Raf kinase inhibitor protein against stroke using ultra-high performance liquid chromatography-quadrupole time-of-flight mass spectrometry (UHPLC-Q-TOFMS), which provides a better understanding of the molecular mechanisms of ischemic stroke and a strong foundation for the development of new therapeutic targets for the treatment of ischemic stroke. In contrast, Liu et al. (2017) used liquid chromatography–mass spectrometry and gas chromatography–mass spectrometry to identify a set of five metabolites to differentiate acute ischemic stroke patients from healthy subjects.

Previous literature has typically focused on the analysis of serum from patients or animals with cerebral ischemia, and there is a relative paucity of studies on feces produced by the body after stroke. However, using feces as a sample to study the association between poststroke metabolism and the GBA after stroke has advantages over the methods described above. For example, fecal samples are non-invasive and easy to collect, and it is easier for researchers to collect large numbers or multiple samples over time to study changes in metabolite levels over time, providing a more complete picture of metabolic activity in the gut of stroke patients. Secondly, due to the protective environment of the gut, fecal metabolites tend to be stable and can be stored for longer periods of time without degradation, improving the stability and accuracy of results and facilitating retrospective analysis (Smith et al., 2020; Erben et al., 2021). Most importantly, fecal metabolites directly reflect the metabolic activity of the gut microbiome, facilitating direct interpretation of the link between GBA and stroke pathogenesis (Zierer et al., 2018; Xu et al., 2019). Not only that, Zhao et al. (2022) analysed feces, urine and plasma from ischaemic stroke patients and healthy groups using a combination of non-targeted metabolomics and macrogenomics, and found that fecal metabolites provided the richest metabolic information and showed the strongest correlation with the gut microbiome, helping to study the interaction between gut microbiota and metabolites to understand the disease. And there have been many studies using feces as a sample for metabolomics research. For example, Cheema and Pluznick (2019) used untargeted metabolomics to analyse fecal samples to study changes in fecal metabolites of angiotensin II in conventional and germ-free mice. Lupo et al. (2021) used non-targeted metabolomics to analyse stool samples from patients with chronic fatigue syndrome/myalgic encephalomyelitis (CFS/ME) and healthy controls to study the metabolic differences between the two that underlie CFS/ME. Pathogenesis provides insights. Jiang et al. (2021) used untargeted metabolomics to study fecal samples to identify potential post-stroke depression-associated gut microbes and their functional metabolites in rats.

In the present study, we used Sprague–Dawley (SD) rats as subjects through middle cerebral artery occlusion to model cerebral ischemia–reperfusion. UPLC–MS/MS provided us with a method for metabolomic analysis of feces. Further, the purpose of our study was to investigate potential markers in rat feces after ischemic stroke by UPLC-MS/MS, to obtain basic data on changes in markers after ischemic stroke, and ultimately to elucidate the potential link between alterations in metabolic function and stroke. Ultimately, this information will improve our knowledge of stroke disease and help us to identify poststroke biomarkers and relevant targets for stroke therapy.

## 2. Materials and methods

### 2.1. Animals and grouping

Healthy adult male SD rats (300 ± 20 g), Shanghai Laboratory Animals Ltd. provided them for the study (No. SYXK 2020–0002). Rats were housed in a flat area with regulated environmental conditions, including noise levels within 60 dB, temperature between 21 and 25°C, humidity between 40 and 60%, and 12 h of light/12 h of darkness per day(light hours: 7:00–19:00). One week was allowed for rats to acclimatize to the standardized laboratory conditions. All rats were allowed to drink water (autoclaved) and food (60Co-irradiated sterilized breeding chow, No. 2019060676, Beijing Huafukang Biotechnology Co., Ltd.), *ad libitum*, and bedding was changed every other day (60Co irradiated corn cobs, Biotechnology Co., Ltd.) and cages (No. IsoRat900N, Tecniplast Group) every other day. All experimental methods were performed in accordance with guidelines for the care and use of laboratory animals in biomedical research (Jones-bolin, 2012).

The random number table method was used to randomly divide the rats into two groups (*n* = 8/group): (1) the normal group, where the rats were kept without any treatment and under the same conditions; (2) the study group, the rat’s middle cerebral artery was occluded/reperfused byinserting an intracanalized monofilament in the left internal carotid artery of the rat.

### 2.2. Establishment of the study group model

The study group used the Longa method to establish the MCAO/R model (Longa et al., 1989). After 12 h of preoperative fasting, during which time water was freely available, the rats were immobilized in the supine position and anesthetized with 3% isoflurane in 30% O_2_ and 67% N_2_. The skin in the middle of the rats’ necks was prepared and disinfected with alcohol iodine, and a longitudinal neck incision was made along the midline. The left common carotid artery, external carotid artery and internal carotid artery were all directly exposed and isolated after the internal and external sternocleidomastoid muscles were separated. Thereafter, the occipital artery and superior thyroid artery were isolated and cauterized to prevent bleeding with a preheated electrocautery device. The pterygopalatine artery, a branch of the internal carotid artery, was ligated; a lasso was made at the distal end of the external carotid artery. The common and internal carotid arteries were then clamped, and the vessel distal to the external carotid artery was cut between the two knots with a microscopic spinning instrument. The external carotid artery was gently pulled so that it was in the same line as the internal carotid artery, and a marked monofilament (Bosterembolus, Boster Biotechnology Co., Ltd., Wuhan, China)was gently pushed through the incision in the direction of the external carotid artery until resistance was minimized(approximately 18 mm). Finally, the monofilament was secured by ligating the proximal external carotid, and the arterial clips of the common and internal carotid arteries were removed. The monofilament was slowly withdrawn after 90 min of ischemia, and the incision was then sutured and postoperatively disinfected with iodine. Until the rats recovered from the anesthetic, their body temperature was maintained at 37 ± 1°C with an electric blanket during the procedure.

### 2.3. Evaluation of the study group

#### 2.3.1. Scoring of neurological deficits

The neurological deficits were assessed according to the Long five-point scale and were scored as follows (Longa et al., 1989): 0, no signs; 1, the rat was unable to fully extend the forelimb; 2, the rat was paralyzed in one limb and had tail-chasing; 3, the rat could not stand or roll; 4, no spontaneous movement and had impaired consciousness. Scores of 1 to 3 indicated successful modeling. The normal group and the study group were evaluated for neurological symptoms 2 h and 14 days following modeling (see Supplementary Figure S1).

#### 2.3.2. Measurement of cerebral infarct volumes

MRI T2-weighted imaging data were obtained using a 9.4 T MRI Bruker Avance-console and a 35 mm orthogonal volume coil (Pharmascan, microMRI, Bruker Medizintechnik, Germany) to determine the nature of infarction in rats. Scans were performed on each group of rats within 24 h of ischemic stroke. Briefly, isoflurane/O_2_ (3% for induction and 2% for maintenance) was used to anesthetize rats. The rats were maintained in a stable physiological condition by placing them flat on a rodent bed covered with a warm water bath mat. A physiological detector (SurgiVet V3395TPR, Smiths Medical Inc., St Paul, MN) continuously monitored vital signs (temperature, respiration and heart rate).

T2-weighted imaging (T2WI) was acquired using a relaxation enhanced (RARE) sequence with the following parameters: field of view (FOV) = 32 × 32 mm, repetition time (TR) = 2,500 ms, echo time (TE) = 33 ms, average number of averages (averages) = 4, number of layers (slices) = 21, layer thickness (slice thickness) bandwidth = 326,087 Hz, matrix size = 256 × 256, echo spacing (ESP) = 10,000 ms, refocusing angle = 180°, excitation angle = 90°, echo column length (echo train length, ETL) = 8, and k-zero = 3. ITK-SNAP^1^ software was used to segment the images of the cerebral infarct region, and the MATLAB 2013b^2^ program was used to calculate the infarct volumes for each subject. The volume of the infarct was obtained as follows: brain infarct volume = number of pixel points in the brain infarct region× spatial resolution of the pixel points.

### 2.4. LC–MS/MS analysis

#### 2.4.1. Fecal sample collection

Fourteen days after establishment of the study group, fecal samples were collected in the normal and the study group after a 12 h fast. Feces were collected by stress defecation, placed in sterile EP tubes and immediately frozen in liquid nitrogen for 10 min and stored at −80°C until use.

#### 2.4.2. Sample preparation

First, an EP tube was filled with 100 μL of the sample, and 400 μL of 80% precooled methanol in water was added and mixed thoroughly by vortex shaking. The mixture was then placed in an ice bath for 5 min and centrifuged at 15,000 × g for 20 min at 4°C. The supernatant was collected and analyzed by liquid chromatography–mass spectrometry (LC–MS; Barri and Dragsted, 2013). The final sample was lyophilized, resolubilized using an equal volume of cosolvent (methanol:acetonitrile = 1:1), vortexed and centrifuged at 15,000 × g for 20 min at 4°C.Mass spectrometry water was used to dilute an amount of supernatant twice, and the diluted sample was assessed using the machine.

#### 2.4.3. Metabolite detection

On a Vanquish (Thermo Fisher Scientific) ultra-performance liquid chromatograph, the target compounds were separated and chromatographed using a Hypesil Gold column (100 × 2.1 mm, 1.9 μm, Thermo Fisher, United States). Liquid chromatography was performed with mobile phase A containing 5 mM ammonium acetate and 0.1% formic acid (pH = 9.0) and methanol-based mobile phase B. With an injection volume of 1 μL and a flow rate of 0.20 mL/min, the column temperature was set at 40°C. The chromatographic gradient elution program was set up as shown in Table 1; under the control software (Xcalibur, Thermo), the Thermo Q Exactive HFX mass spectrometer was able to acquire primary and secondary mass spectrometry data. The following specific parameters were established: The MS/MS secondary scans are data-dependent scans; capillary temperature is 320°C, sheath gas flow rate is 40 Arb, aux gas flow rate is 10 Arb, spray voltage is 3.2 kV, and polarity is positive and negative.

**Table 1:** Chromatographic gradient elution program.

Time(min)	A%	B%
0	98	2
1.5	98	2
12	0	100
14	0	100
14.1	98	2
17.0	98	2

#### 2.4.4. Data extraction and multivariate analysis

For peak detection and alignment, sample data were extracted using Compound Discover (CD) software (version V3.1, Thermo Fisher, Germany). Internal standard normalization was used in the examination of the data, and the final data set, including peak number information, sample name and normalized peak area was then imported into SIMCA software (V16.0.2, Sartorius Stedim Data Analytics AB, Umea, Sweden) for principal component analysis (PCA) of fecal metabolites (Wiklund et al., 2008) and orthogonal partial minimum analysis (OPM). Pattern recognition analysis was performed by orthogonal projections to latent structures-discriminant analysis (OPLS-DA). Metabolites with a variable importance in the projection (VIP) greater than 1 and *p* < 0.05 were considered differential metabolites (Saccenti and Hoefsloot, 2014). Potential biomarkers were found using the Kyoto Encyclopedia of Genes and Genomes (KEGG) database^3^ and the Human Metabolome Database (HMDB).^4^ In addition, pathway enrichment analysis was performed using many databases, including PubChem^5^ and MetaboAnalyst.^6^

### 2.5. Statistical analysis

Excel 2019 for Windows (Microsoft Corporation, San Francisco, CA, United States) was used to generate the database, and IBM SPSS version 24.0 statistical software (SPSS Inc., Chicago, IL, United States) was utilized for all statistical analyses. The normality of the distributions of continuous variables was tested, with normally distributed continuous variables expressed as the mean ± standard deviation (SD); the means of continuous normally distributed variables were compared by independent samples Student’s *t* test. Nonnormal variable medians were compared using nonparametric testing. Differences were considered statistically significant if *p* < 0.05.

## 3. Results

### 3.1. Neurological deficit scores and imaging evaluation in model rats with cerebral ischemia stroke

**Figure 1:**
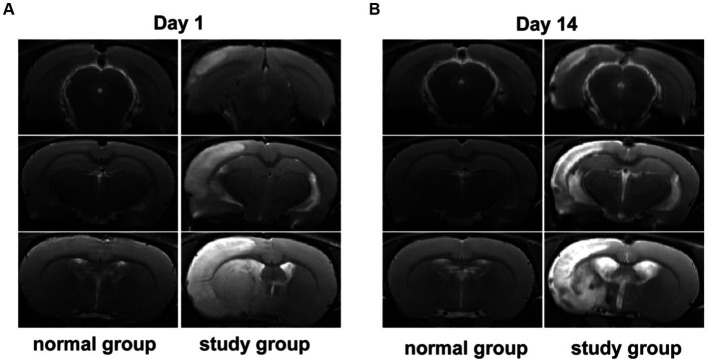
**(A,B)** Show the signal changes in the normal group and the study group at day 1 and day 14 after brain infarction.

The rats in both groups were scored for neurological deficits at each time point, and the results are present in Table 2. At each time point, the rats in the normal group did not show any neurological deficits, whereas the study group showed different degrees of neurological deficits at 1 and 14 days of ischemic stroke. A statistically significant (*p* < 0.001) difference in scores was noted compared to the normal group. The infarcts of the rats were assessed at 1 and 14 days after ischemic stoke injury using T2WI structural images at the specific infarct locations shown in Figure 1, including brain regions such as the hippocampus, internal olfactory cortex, motor cortex, sensory cortex, dorsal thalamus and striatum. As shown in Table 3, the infarct volume increased more significantly in the study group of rats at different time points 1 day and 14 days after modeling (1 day, *p* < 0.001; 14 day: *p* = 0.001). In conclusion, these results provide evidence that the rat model used in this study was stable.

**Table 2:** Comparison of longa scores between rat groups.

Group	*n*	1d	14 d
normal group	8	0	0
Study group	8	2.50 ± 0.54	1.38 ± 0.27
Z		−3.651	−3.664
*P*		<0.001	<0.001

**Table 3:** Comparison of the percentage of cerebral infarct volume in each group of rats.

Group	*n*	1d	14 d
Normal group	8	0	0
Study group	8	20.90 ± 4.50	13.85 ± 3.65
t		13.131	10.724
*P*		<0.001	0.001

### 3.2. Nontargeted metabolomics results

#### 3.2.1. Quality control results

**Figure 2:**
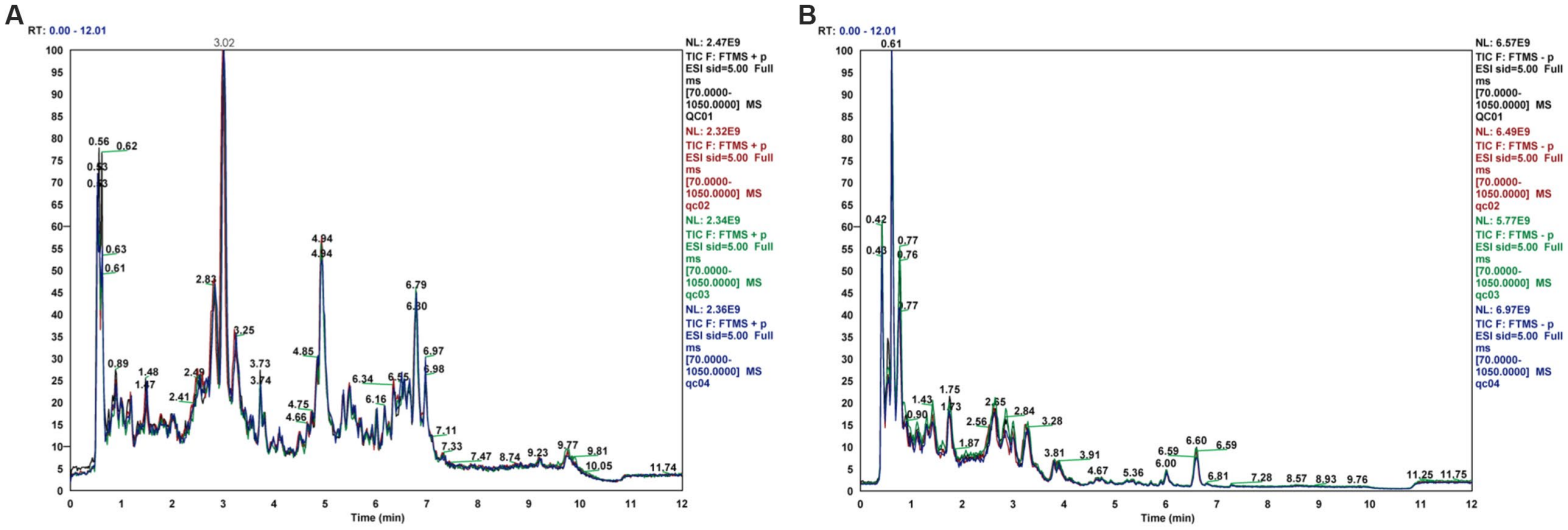
Total ion current diagram for rat fecal samples in positive and negative ion modes, **(A)** Total ion current diagram (ESI+), **(B)** Total ion current diagram (ESI-).

The stability of the assay can be judged by the difference in response peak heights of the internal standards between the quality control samples. The extracted ion current (EIC) plots for all samples are shown in Figures 2A,B. The results indicate that the response intensities and retention periods of the peaks basically overlap, revealing that the variation caused by instrument error is minimal throughout the process. The response peak heights of all samples almost overlap, and the peak heights, peak areas and retention times are approximately the same, indicating good stability of the instrument data acquisition.

#### 3.2.2. Results of multivariate statistical analysis of fecal metabolomics

**Figure 3:**
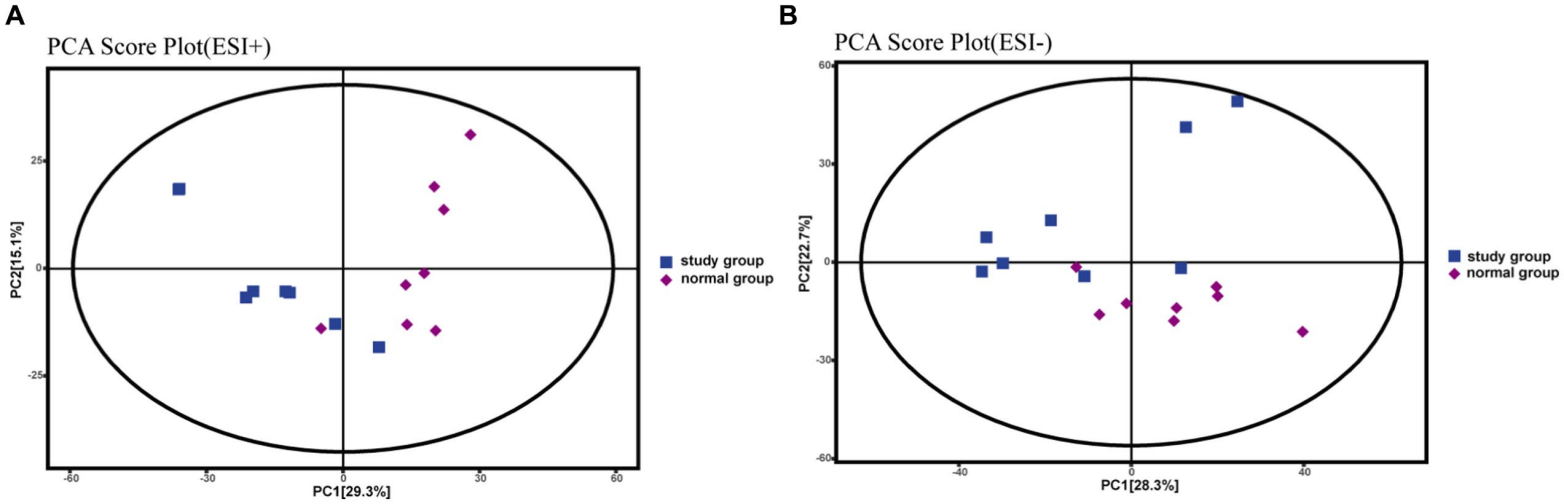
PCA score plots for rat fecal metabolomics, **(A)** PCA Score Plot (ESI+), **(B)** PCA Score Plot (ESI-).

PCA is an unsupervised statistical method that uses an orthogonal transformation to turn a set of observed potentially correlated variables into linearly uncorrelated variables (i.e., principal components), whereas OPLS-DA is a supervised statistical method that allows for repeated analysis of samples between groups, allowing for more reliable group differences in metabolites. The PCA score plot (Figures 3A,B) shows that all samples fell within the 95% confidence interval (Hotelling’s T-squared ellipse) and that the normal and model groups were separated in both positive and negative ion modes, indicating that the differences between the two groups’ metabolites are displayed.

**Figure 4:**
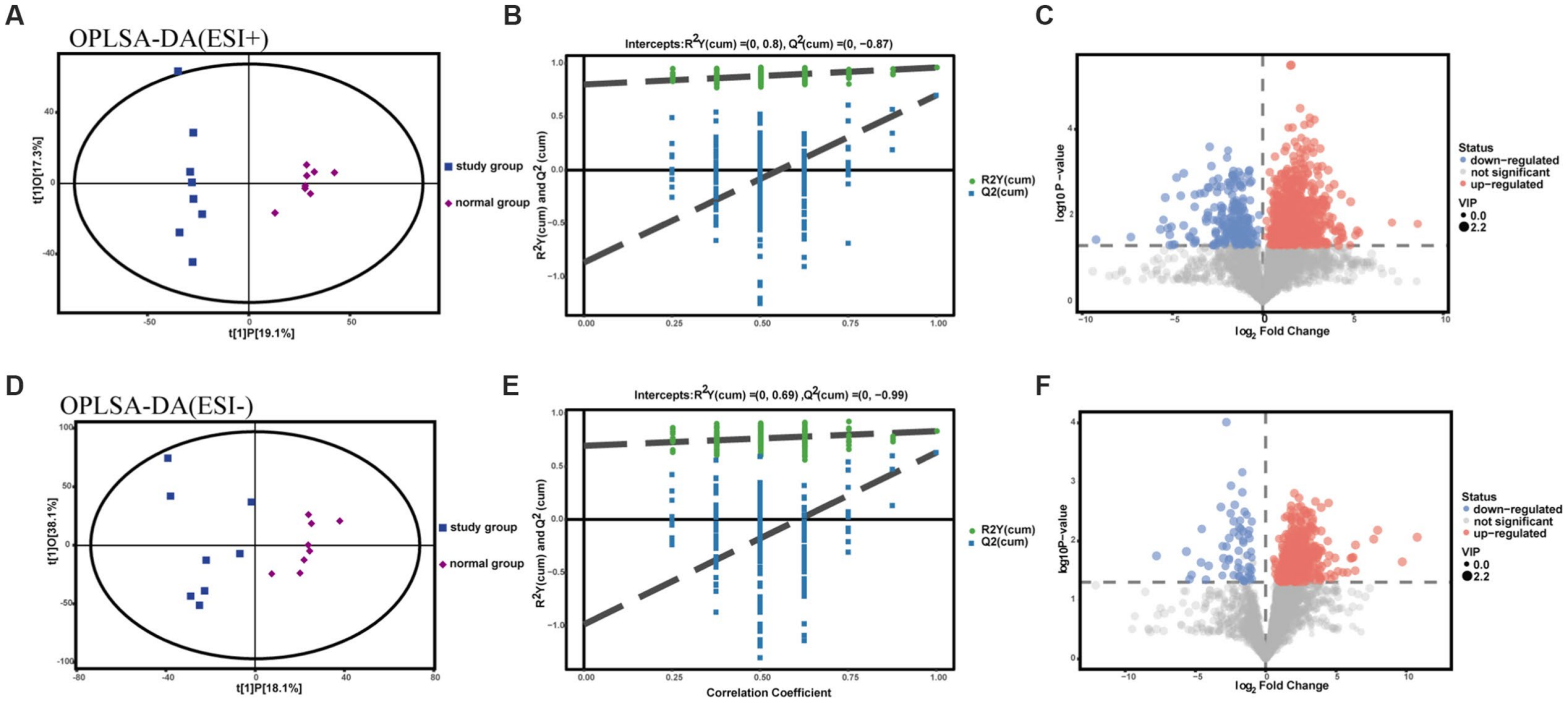
OPLS-DA plot, substitution test plot and volcano plot for metabolomic analysis of rat feces. **(A-C)** ESI+ model. **(D-F)** ESI- model.

To assess intergroup differences in fecal metabolites between the normal and studyrat groups, OPLS-DA modeling analysis was performed using SIMCA 16.0.2 software, and model quality was tested using a 7-fold cross-validation test. In both positive and negative ion identification modes, scatter plots of the initial OPLS-DA model scores showed between-group differences between the normal model and studyrat fecal metabolome groups (Figures 4A,D). The R^2^Y (to assess the interpretability of the categorical variable Y) and Q^2^ (to assess the predictability of the model) values were recorded for each of the 200 permutation tests performed on the model (Figures 4B,E). This finding indicates that there is no overfitting in the initial OPLS-DA model, the two models established are more in line with the actual situation of the sample data, and the original model can reasonably explain the differences between the two sample groups. Therefore, the model has good adaptability and predictability. On this basis, volcano plots were used to display the metabolites that were significantly different between the normal group and the model group (Figures 4C,F).

#### 3.2.3. Biomarker screening and pathway enrichment analysis

The compounds identified by the positive and negative ion patterns were deredundantly integrated and screened for differential metabolites. According to the above rules, this study’s cardinality criterion was a *p* value of less than 0.05 for Student’s t test, whereas the OPLS-DA model’s first principal component’s VIP was more than 1 to screen for differentially expressed metabolites in the two groups. The model and normal groups identified 1,044 differential metabolites in the positive ion model and 635 in the negative ion model. Due to the high-throughput nature of the Q-Exactive (QE) metabolome data, a large number of distinct metabolites were detected in this study, so only differential metabolites with a cardinality criterion of *p* < 0.05 and a VIP ≥ 2 are listed in this study. Table 4 provides a summary of the basic characteristics of these differential metabolites.

**Table 4:** Identification of two groups of differential metabolites.

ID	MS2 name	Mass(m/z)	R.T.(min)	VIP	*p*	Change trend
308	Niazicinin A	370.14868975987	427.005	2.045006567	0.00324	↑
837	N-Methyl-1-deoxynojirimycin	178.107096619874	121.976	2.018372203	0.019344	↓
115	Isogenistein 7-glucoside	433.11163285909	116.655	2.022836	0.001486	↓
*20*	Creatinine	114.066252904115	179.566	2.020235	0.018372	↓
*292*	5′-Methylthioadenosine	296.081080810132	382.4415	2.006154	0.004204	↑

**Figure 5:**
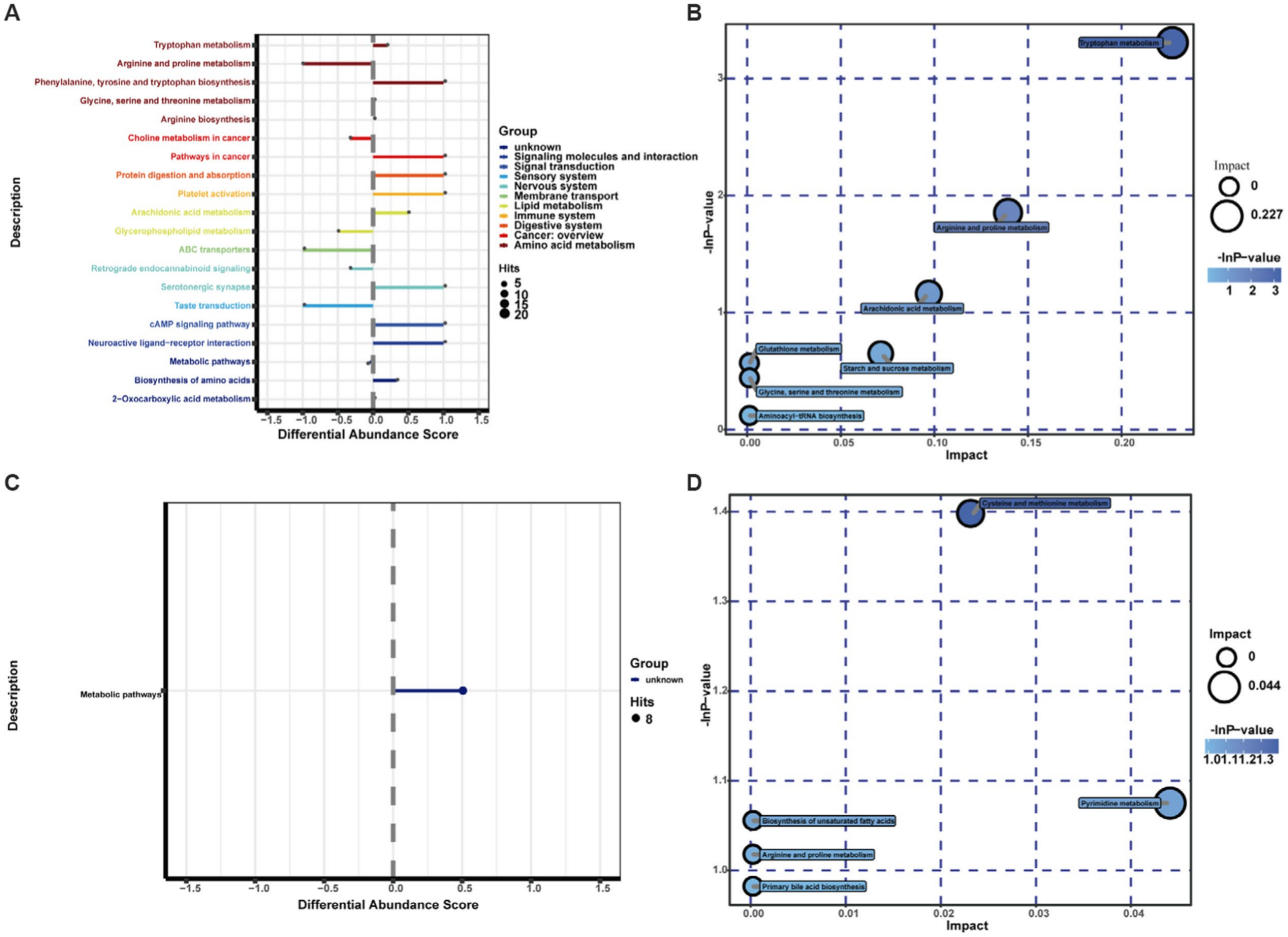
Differential pathway scores and metabolic pathway enrichment analysis bubble diagrams for the study group and normal group in positive and negative ion mode: the different colors of the pathways in **(A,C)** indicate the different metabolic classifications to which the pathways belong; the line segments indicate the up- and downregulation of the pathways; positive values of the line segments indicate the overall upregulation of the pathways; conversely, negative values of the line segments indicate the overall downregulation of the pathways; the size of the line endpoints indicates the amount of material annotated in the pathway. Each bubble in **(B,D)** represents a metabolic pathway. The horizontal coordinate of the bubble and the size of the bubble indicate the influence factor of the pathway in the topological analysis; the larger the size, the larger the influence factor. The vertical coordinate of the bubble and the color of the bubble indicate the *p* value [negative natural logarithm, i.e., -In(p)] of the enrichment analysis. The darker the color, the smaller the *P* value, and the more significant the enrichment.

The KEGG IDs of the differential metabolites were then uploaded to MetaboAnalyst for visual analysis and KEGG pathway analysis. The differential abundance score in the KEGG pathway difference abundance analysis is an analysis method based on the metabolic changes in the pathway, which can intuitively display the up- and downregulation of the metabolic pathway and the metabolic type (Figures 5A,C). According to the results, tryptophan metabolism, arginine and proline metabolism, arachidonic acid metabolism, starch and cross metabolism, cysteine and metabolism, and pyrimidine metabolism, are all closely associated with cerebral ischemic stroke injury. Among them, the metabolic pathways upregulated after cerebral ischemia/reperfusion injury included tryptophan metabolism, arachidonic acid metabolism, cysteine and methyl metabolism, and pyrimidine metabolism, and the metabolic pathways downregulated were arginine and proline metabolism and starch and cross metabolism. Through exhaustive analysis of the differential metabolite pathways (including enrichment analysis and topology analysis), we found the key pathway with the highest correlation with the difference in metabolites (Table 5; Figures 5B,D).The difference in metabolites between the normal group rats and the study group rats was mainly related to the tryptophan metabolic pathway, according to the data.

**Table 5:** Results of integrated enrichment analysis of biomarkers using MetaboAnalyst.

	Pathway name	Total	Hits	Raw *p*	-ln(p)	Impact
1	Tryptophan metabolism	41	4	0.036678	3.3056	0.22718
2	Arginine and proline metabolism	44	3	0.15695	1.8518	0.13934
3	Arachidonic acid metabolism	36	2	0.31307	1.1613	0.09707
4	Starch and sucrose metabolism	23	1	0.52256	0.64902	0.07135
5	Cysteine and methionine metabolism	28	1	0.24705	1.3981	0.02313
6	Pyrimidine metabolism	41	1	0.3413	1.075	0.04412

## 4. Discussion

The development of stroke significantly alters the synthesis and molecular metabolism of body substances, and bidirectional communication between the brain and peripheral organs is important in patients after stroke (Iadecola and Anrather, 2011). Gut microbial colonization plays a significant role in the development of the host gut immune response (Gensollen et al., 2016). In contrast, differential metabolites in the pre- and poststroke rat gut reflect pathophysiological processes, such as energy deficit, inflammation, neurotoxicity, oxidative stress, neuroexcitation and injury, and are biochemical indicators that can be used to evaluate physiological and pathological processes (Zhang et al., 2021). Therefore, quantitative metabolomic analysis of pre- and poststroke metabolic changes in rats can increase the understanding of disease pathogenesis and improve the development of treatment protocols. We replicated the MCAO/R model using the Longa method and found that the MCAO/R rats not only had significant neurological deficits but also a significant increase in infarct volume in the brain after 1 and 14 days of modeling using neurological deficit scores and T2WI structural images. This provides further evidence of the stability of the MCAO\R model used in this study. Afterwards, we used UPLC–MS/MS metabolomics to perform a comprehensive study of metabolites in the feces of rats in an ischemic stroke model to provide an overall picture of metabolite changes. According to the PCA plots, 1,044 and 635 differential metabolites connected to stroke were identified in rat feces in positive and negative ion mode, respectively, separating the study group from the normal group in terms of potential biomarkers. Then, KEGG pathway analysis was performed using MetaboAnalyst, which revealed the presence of poststroke metabolites in rats. Stroke in rats causes abnormalities in the metabolism of a variety of substances, including tryptophan, arachidonic acid, cysteine and methionine, pyrimidine, arginine and proline, starch, and sucrose, suggesting that ischemic brain injury leads to metabolic disorders in the above six metabolic pathways in rats. Specifically, tryptophan metabolism was the most affected.

As mentioned earlier, brain injury due to ischemic stroke is accompanied by neuropathological events, such as oxidative stress and neuroinflammation. In contrast, arachidonic acid is a precursor of proinflammatory mediators and has a crucial role in the development of inflammation. Arachidonic acid is metabolized by the lipoxygenase, cyclooxygenase and cytochrome P450 epoxygenase pathways to produce a variety of active metabolic small molecules, which have a role in regulating the intensity and duration of the inflammatory response and are key regulators of oxidative stress and inflammation (Medzhitov, 2008; Alhouayek and Muccioli, 2014; Zhang et al., 2020). Methionine is crucial for the brain during and after hypoxia and is also a powerful antioxidant, as the sulfhydryl groups contained in methionine scavenge free radicals and have some antioxidant capacity. Thus, methionine plays a crucial role in limiting the level of reactive oxygen radicals, both directly and indirectly (Flores et al., 2013). In addition, methionine and its related derivatives protect the brain from the damaging effects of hypoxia (Burgess et al., 2020). Methionine is involved not only in one-carbon unit metabolism and methylation reactions but also in the synthesis and repair of various DNA strands and in the expression of genes, the regulation and stabilization of protein function and the processing of ribonucleic acids (Goulart et al., 2019), thereby enhancing pyrimidine metabolism. Methionine metabolism produces cysteine, which synthesizes glutathione, a key antioxidant molecule involved in protein translation (Elango, 2020). Therefore, the upregulation of cysteine and methionine metabolic pathways is the main complementary pathway providing raw material for glutathione synthesis in mammals, enhancing antioxidant action *in vivo*. In turn, arginine, similar to many antioxidants, enhances the body’s antioxidant capacity and improves systemic immune function (Mm et al., 2014). Nitric oxide (NO) produced by arginine metabolism has an important role in cardiovascular regulation. NO regulates blood flow, relaxes blood vessels and inhibits platelet aggregation, which facilitates recovery from stroke-related dysfunction (Conti et al., 2013). Arginine is a precursor to proline and creatine, as well as the amino acid with the largest nitrogen-donating capacity (Ge et al., 2020). The metabolism of proline produces electrons and reactive oxygen species, which cause a number of downstream effects, such as blocking the cell cycle, autophagy and apoptosis (Selen et al., 2015). Finally, when the onset of acute cerebral ischemia is followed by the inhibition of oxidative phosphorylation during rapid oxygen dissipation in brain tissue, a dramatic reduction in brain tissue ATP levels within only a few seconds leads to energy metabolic failure and apoptotic necrosis of neurons, allowing for the downregulation of starch and sucrose metabolism, which subsequently affects the body’s energy metabolism (Heiss, 2012). Furthermore, mitochondrial DNA encodes genes related to ATP synthesis, and downregulation of their expression results in a decrease in ATP production (Ji et al., 2018). Finally, tryptophan is an inhibitor of gluconeogenesis (Barik, 2020). The above multiple causes contribute to the downregulation of starch and sucrose metabolism.

In contrast, tryptophan and its metabolites are essential for the maintenance of neurological function, immunity and homeostasis *in vivo*, in addition to being the building blocks of protein synthesis. Tryptophan metabolites are implicated in the pathological processes of a variety of diseases, including immune responses, and exert different biological effects in the regulation of many diseases (Comai et al., 2019). Tryptophan metabolism is involved in the regulation of the GBA, and the gut flora can mediate the regulation of the kynurenine pathway by tryptophan through direct and indirect mechanisms (Kennedy et al., 2017). Tryptophan metabolites, including 5-hydroxytryptophan, kynurenine, tryptamine and indole compounds, have profound effects on the interactions between the gut microbiota and the GBA (Clarke et al., 2012; Rothhammer et al., 2016).

Tryptophan metabolism can be broadly divided into three pathways: (1) the kynurenine pathway, (2) the 5-hydroxytryptamine pathway, and (3) Indole pathway for gut flora (Tomberlin et al., 2016; Wang et al., 2017). Over 95% of total tryptophan in the host is oxidized to kynurenine via the kynurenine pathway (Cervenka et al., 2017). It is believed that the kynurenine pathway plays a crucial role in the regulation of neurotransmission and immune function. In its pathway production, increased inflammation or oxidative stress can activate the activity of rate-limiting enzymes associated with induction (Jiang et al., 2018). Meanwhile, microbial regulation of the kynurenine pathway is similarly regulated by inflammatory mediators and is immunoreactive (Kennedy et al., 2017). The kynurenine pathway is present in many tissues (mainly the liver, brain and gut) (Fernstrom, 2016). In addition, intestinal flora can directly utilize tryptophan, with 4–6% of tryptophan being metabolized by intestinal flora (Wang et al., 2019). Poststroke inflammation induces upregulation of the kynurenine pathway of tryptophan oxidation, leading to neuroprotection (kynurenine acid) and neurotoxic metabolites (quinolinic acid). Wang et al. (2017) used a metabolomics approach to examine the serum metabolic profile between acute ischemic stroke patients and controls and observed reduced tryptophan levels, again demonstrating enhanced tryptophan metabolism. Brouns et al. (2010) found that poststroke inflammation induced upregulation of the kynurenine pathway of tryptophan oxidation by measuring plasma tryptophan and its metabolite concentrations at admission, 24 h, 72 h and day 7 in 149 stroke patients，and has a neuroprotective effect on the organism. Therefore,tryptophan metabolism via the kynurenine pathway is associated with immunity and inflammation and that blocking the associated pathway may prevent inflammatory responses after stroke. Most central nervous system (CNS) kynurenine is of peripheral origin, and once in the CNS, it can also be involved in subsequent metabolism (Gheorghe et al., 2019). The kynurenine pathway produces kynurenine, which is further metabolized by two different pathways: neuroprotective quinolinic acid and kynurenine (Cervenka et al., 2017). These metabolites from the kynurenine pathway, known as “kynurenines,” not only act as mediators of inflammation but also cross the blood–brain barrier to reach the central nervous system. Kynurenines are therefore regarded as neuromodulators in numerous physiological and pathological processes of brain and gastrointestinal dysfunction (Kennedy et al., 2017). Not only that, studies have shown that the kynurenine pathway is more active in acute stroke patients. Darlington et al. (2007) demonstrated that acute stroke patients rapidly activate the kynurenine pathway. In contrast, blocking the kynurenine pathway was found to reduce infarct volume in animal models (Cozzi et al., 1999) 0.5-Hydroxytryptamine, also known as serotonin, and tryptophan, the only precursor of serotonin, are important for neurotransmission. Serotonin not only regulates the immune activity of the body but also serves as a critical neurotransmitter in the GBA (Agus et al., 2018) and is involved in a wide range of human physiological functions by activating specific serotonin receptors (Israelyan and Margolis, 2018). However, central serotonin accounts for only a small fraction of the body’s total serotonin. Over 90% of serotonin is located in the gastrointestinal tract (Fouquet et al., 2019). Melatonin is mainly produced by the catabolism of serotonin and is a free radical scavenger that has strong antioxidant and anti-inflammatory effects (Gunata et al., 2019; Zhao et al., 2019). The article by Lian et al. (2023) found that inducing melatonin production by the gut microbiota could promote gut homeostasis, improve gut barrier function, and ultimately reduce brain and gut damage, thereby preventing ischaemic stroke.

Finally, tryptophan can be metabolized into a variety of metabolizable indole derivatives, which are mainly produced by gut microbes and are part of the kynurenine pathway. As these indole metabolites are ligands for aromatic hydrocarbon receptors, they are considered to be important components of the immune response (Alexeev et al., 2018). Not only that, but these metabolites play an important role in maintaining gut homeostasis and systemic immunity, and may also influence disease development and progression (Su et al., 2022; Ye et al., 2022). Importantly, studies have shown that related indole derivatives can significantly reduce cellular oxidative stress, inflammation and neuronal apoptosis (Yin et al., 2023). Wei et al. (2021) found that indole derivatives produced by gut microbes can enhance neurogenesis in mice by promoting the differentiation of neural progenitor cells into neurons via aryl hydrocarbon receptors. Indole treatment also increased neurite outgrowth and synaptogenesis in mice.

## 5. Conclusion

In this study, we demonstrated that metabolomics is an experimental approach to explore metabolic pathways in poststroke rats after stroke and that differences in fecal metabolites in rats 14 days after stroke are most evident in the disruption of tryptophan metabolism. Therefore, future interventions to study the treatment of stroke could target the tryptophan pathway in the gut microbiome or promote the beneficial products of tryptophan metabolism as one of the targets of intervention.

## 6. Limitation

We need to study metabolic changes in the brain tissue of stroke rats in the future to further demonstrate the role of metabolism, such as tryptophan, in the development of stroke. Studies have shown known sex differences in tryptophan metabolism in healthy adults or rats, with females having higher tryptophan metabolism or tryptophan utilization than males (Leskanicova et al., 2020; Cai et al., 2021; Pais et al., 2023), which may limit the translation of our study. Finally, we will invest in further studies in the future to determine whether tryptophan metabolism in female rats versus patients in the clinical setting has the same results as noted in this study.

## Data availability statement

The original data presented in the study is included in the article/supplementary material, further inquiries can be directed to the corresponding authors.

## Ethics statement

The animal study was reviewed and approved by Ethics Committee of Fujian University of Fujian University of Traditional Chinese Medicine.

## Author contributions

AZ and ZL conceived and designed the research. DH and YZ conducted the experiments. YY and WS collected the data. CJ analyzed the data. XK and YY wrote the manuscript. All authors contributed to the article and approved the submitted version.

## Funding

This work was supported by the Research Start-Up Special Foundation of Shanghai Fourth People’s Hospital Affiliated to Tongji University (Grant No. sykyqd02001) and Discipline Booster Program of Shanghai Fourth People’s Hospital Affiliated to Tongji University (Grant No. sy-xkzt-2022-1004).

## Conflict of interest

The authors declare that the research was conducted in the absence of any commercial or financial relationships that could be construed as a potential conflict of interest.

The reviewer ZJ declared a shared affiliation with the author(s) XK to the handling editor at the time of review.

## Publisher’s note

All claims expressed in this article are solely those of the authors and do not necessarily represent those of their affiliated organizations, or those of the publisher, the editors and the reviewers. Any product that may be evaluated in this article, or claim that may be made by its manufacturer, is not guaranteed or endorsed by the publisher.

## Footnotes

^1^http://www.itksnap.org/pmwiki/pmwiki.php

^2^https://www.mathworks.com

^3^http://www.genome.jp/kegg/

^4^https://www.hmdb.ca/

^5^https://www.ncbi.nlm.nih.gov/pccompound

^6^http://www.metaboanalyst.ca/
