# Supplementary figures and images for: Untargeted metabonomic analysis of a cerebral stroke model in rats: a study based on UPLC–MS/MS
Source: Front Neurosci. 2023 Aug 8;17:1084813. doi: 10.3389/fnins.2023.1084813 (PMC10442664; doi:10.3389/fnins.2023.1084813)

***Supplementary Material***


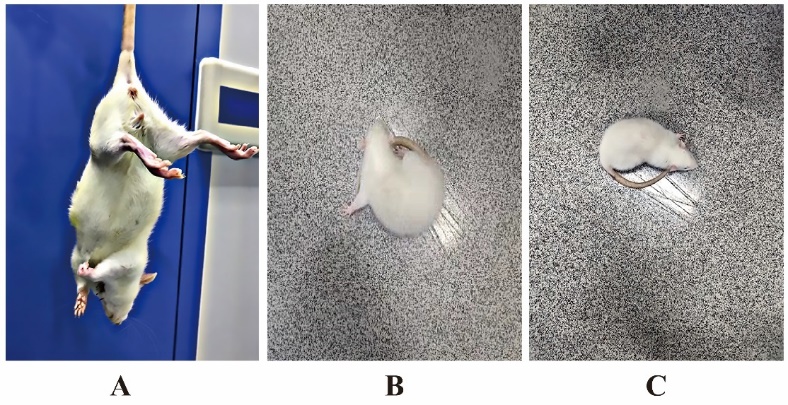


Supplementary Figure S1: A: score 1 points; B: score 2 points; C: score 3 points

Supplement: Supplementary file 1 [file Table_1.DOCX]
